# De Novo Origins of Human Genes

**DOI:** 10.1371/journal.pgen.1002381

**Published:** 2011-11-10

**Authors:** Daniele Guerzoni, Aoife McLysaght

**Affiliations:** Smurfit Institute of Genetics, University of Dublin, Trinity College, Dublin, Ireland; University of California Davis, United States of America

Where do new genes come from? For a long time the answer to that question has simply been “from other genes”. The most prolific source of new loci in eukaryotic genomes is gene duplication in all its guises: exon shuffling, tandem duplication, retrocopying, segmental duplication, and genome duplication. However, in recent years there has been a growing appreciation of the oft-dismissed possibility of evolution of new genes from scratch (i.e., de novo) as a rare but consistent feature of eukaryotic genomes [1], [2].

Pioneering work identified several de novo genes in *Drosophila* [3]–[5], and since then, additional *Drosophila* cases have been identified [6], as well as cases in yeast [7], [8], *Plasmodium* [9], rice [10], mouse [11], primates [12], and human [13], [14]. It would appear that whenever anyone makes the effort to search, candidate novel genes are found.

In this issue of *PLoS Genetics*, Wu et al. [15] report 60 putative de novo human-specific genes. This is a lot higher than a previous, admittedly conservative, estimate of 18 such genes [13], [16]. The genes identified share broad characteristics with other reported de novo genes [13]: they are short, and all but one consist of a single exon. In other words, the genes are simple, and their evolution de novo seems plausible. The potential evolution of complex features such as intron splicing and protein domains within de novo genes remains somewhat puzzling. However, features such as proto-splice sites may pre-date novel genes [9], [17], and the appearance of protein domains by convergent evolution may be more likely than previously thought [2].

The operational definition of a de novo gene used by Wu et al. [15] means that there may be an ORF (and thus potentially a protein-coding gene) in the chimpanzee genome that is up to 80% of the length of the human gene (for about a third of the genes the chimpanzee ORF is at least 50% of the length of the human gene). This is a more lenient criterion than employed by other studies, and this may partly explain the comparatively high number of de novo genes identified. Some of these cases may be human-specific extensions of pre-existing genes, rather than entirely de novo genes—an interesting, but distinct, phenomenon.

## Limitations in Defining and Identifying De Novo Genes

A major consideration in these studies is the reliable definition and identification of de novo genes. If a sequence similarity search fails to return a plausible homolog, then it may be that you are dealing with a novel gene. However, it is necessary to exclude the alternative hypothesis of recent loss in sister lineages as well as the possibility that this is a rapidly evolving gene with highly divergent, but extant, homologs.

**Figure 1:**
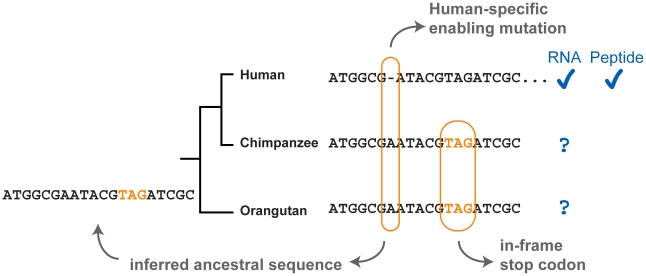
Evidence in the detection of novel genes. A hypothetical example where a novel human ORF is created by a human-specific deletion. The 1 bp deletion shifts a downstream stop codon out of frame. Because the deletion is not shared by other primates, the ancestral sequence is inferred to carry the in-frame stop. The authenticity of the novel human gene can be confirmed with transcription and translation evidence.

Wu et al. [15] have employed a strategy similar to that of Knowles and McLysaght [13] to search within the human genome for candidate novel loci. The search protocol requires positive evidence of the absence of the gene from other primate lineages in order to show that it is not a gene that has diverged beyond recognition from its homologs (orthologous DNA is identifiable), nor is it a gene that has been recently lost in sister lineages (the ancestral sequence is inferred to carry a disablement) (Figure 1).

A serious limitation in this approach is that it relies on existing gene lists that have been annotated using criteria that usually include the presence of a homolog in other genomes. Novel genes fail to meet this criterion by definition, thus they are usually not reliably annotated. Wu et al.'s study [15] highlights the volatility of the annotation of putatively novel genes—over half of the candidate de novo genes they identified are not included in the more recent Ensembl gene lists they used (version 56), and by version 60 only six of these genes were still listed.

It would be preferable to have a method of identifying novel genes that used more direct evidence of gene expression. Sequenced peptides and ESTs can be used to confirm that a putative gene is operational, but these data are not currently suitable for identifying protein-coding genes from first principles: the peptide databases usually only list peptides belonging to already-annotated genes [18]; and the high rate of promiscuous transcription of the genome, particularly in testis, where several of Wu et al.'s genes [15] were expressed at their highest, means that transcription alone is not sufficient to recognize a gene [1], [19].

However, care must also be taken to ensure that the ancestral sequence can reliably be inferred to be non-coding. Wu et al. [15] restricted their search to chimpanzee and orangutan genomes, but in at least one case (ENSG00000221972) gorilla and gibbon share the “human-specific” mutation, making this case equivocal. Ideally, the putative non-coding sequences should be investigated for evidence of transcription and translation to support the inference of absence of coding capacity.

## Future Challenges

Though Wu et al. [15] have contributed to our growing knowledge of de novo gene evolution, we still lack a definitive list of de novo–originated genes in the human genome—mainly due to issues concerning genome annotation and the stringent criteria required to reliably identify cases. A comprehensive list of de novo genes in human as well as in other primates would open up the opportunity to examine the survivorship of these genes and investigate their specific contribution to phenotype.

The observation by Wu et al. [15] that some of the candidate de novo genes are expressed at their highest in brain tissues and testis is interesting, but by no means proves they are functional. A major challenge remains to demonstrate functionality of the de novo genes. This is particularly difficult for human-specific genes, where there is perhaps the greatest interest, but there are also the greatest limitations in terms of possible experiments.

## What Does This Tell Us about Human–Chimpanzee Divergence?

Though it remains to be seen if any of the genes is functional, a clear picture is developing of de novo evolution as a process that can create genetic novelty, upon which there is at least the *opportunity* for natural selection to act. It has been argued that the capacity for innovation generated by novel genes is particularly important for the evolution of lineage-specific traits [20].

It is now common knowledge that human and chimpanzee DNA differ by only 1% (more accurately, they differ in 1% of alignable regions of genome, with a further 3% divergence due to lineage-specific indels [21]). This fact lies in stark contrast to the large phenotypic differences between the two species [22]. The study by Wu et al. [15], along with the previous reports of de novo genes in human, shows that even within highly similar regions of DNA, we can pinpoint small changes at the nucleotide level—base substitutions and indels—that have the potential to generate large phenotypic effects.
